# A mechanism for matrikine regulation in acute inflammatory lung injury

**DOI:** 10.1172/jci.insight.140750

**Published:** 2021-04-08

**Authors:** Sarah W. Robison, JinDong Li, Liliana Viera, Jonathan P. Blackburn, Rakesh P. Patel, J. Edwin Blalock, Amit Gaggar, Xin Xu

**Affiliations:** 1 Department of Medicine, Division of Pulmonology, Allergy and Critical Care Medicine, and; 2 Program in Protease and Matrix Biology, University of Alabama at Birmingham, Birmingham, Alabama, USA.; 3 Birmingham VA Medical Center, Birmingham, Alabama, USA.; 4 Department of Pathology, Division of Molecular and Cellular Pathology, and; 5 Center for Free Radical Biology, University of Alabama at Birmingham, Birmingham, Alabama, USA.; 6 Gregory Fleming James Cystic Fibrosis Research Center, Birmingham, Alabama, USA.; 7 Lung Health Center, University of Alabama at Birmingham, Birmingham, Alabama, USA.

**Keywords:** COVID-19, Inflammation, Extracellular matrix, Neutrophils, Peptides

## Abstract

Proline-glycine-proline (PGP) and its acetylated form (Ac-PGP) are neutrophil chemoattractants generated by collagen degradation, and they have been shown to play a role in chronic inflammatory disease. However, the mechanism for matrikine regulation in acute inflammation has not been well established. Here, we show that these peptides are actively transported from the lung by the oligopeptide transporter, PEPT2. Following intratracheal instillation of Ac-PGP in a mouse model, there was a rapid decline in concentration of the labeled peptide in the bronchoalveolar lavage (BAL) over time and redistribution to extrapulmonary sites. In vitro knockdown of the PEPT2 transporter in airway epithelia or use of a competitive inhibitor of PEPT2, cefadroxil, significantly reduced uptake of Ac-PGP. Animals that received intratracheal Ac-PGP plus cefadroxil had higher levels of Ac-PGP in BAL and lung tissue. Utilizing an acute LPS-induced lung injury model, we demonstrate that PEPT2 blockade enhanced pulmonary Ac-PGP levels and lung inflammation. We further validated this effect using clinical samples from patients with acute lung injury in coculture with airway epithelia. This is the first study to our knowledge to determine the in vitro and in vivo significance of active matrikine transport as a mechanism of modulating acute inflammation and to demonstrate that it may serve as a potential therapeutic target.

## Introduction

Acute lung injury (ALI) is a clinical syndrome, with acute respiratory distress syndrome (ARDS) at the severe end of the disease spectrum, defined by bilateral noncardiogenic pulmonary infiltrates with hypoxemic respiratory failure (1). This critical illness remains a major cause of morbidity and mortality in the ICU (2, 3). The disease is characterized by acute and progressive neutrophil-mediated lung inflammation, with tissue remodeling leading to loss of adequate gas exchange (4). While recent improvements in the management of ARDS, such as low tidal volume ventilation and prone positioning (5, 6), have improved mortality, there are no therapeutic interventions that target mechanisms/mediators underlying the pathobiology of this disease.

A key feature of ARDS is proteolytic degradation of the extracellular matrix that leads to organ remodeling and defects in lung ventilation/perfusion (7–9). This tissue degradation leads to the generation of matrikines, bioactive extracellular matrix fragments that affect cell behavior (10, 11). Matrix-derived bioactive peptides are increasingly appreciated as critical mediators of inflammatory tissue injury in chronic lung disease (12).

The collagen-derived matrikine, proline-glycine-proline (PGP), and its more potent acetylated variant (Ac-PGP) act as a mimetic of CXCR1 and CXCR2 ligands, leading to PMN recruitment and activation in the inflamed lung (13). We have shown increased levels of these peptides in patients with COPD (14), cystic fibrosis (15), neutrophilic asthma (16), and bronchiolitis obliterans syndrome after lung transplantation (17), and, in all of these conditions, the elevation of these peptides corresponds to key disease-related metrics (18).

A discrete proteolytic pathway has been characterized for PGP peptide generation (15) and in chronic inflammation; the enzyme leukotriene A4 hydrolase and angiotensin-converting enzyme have been shown to degrade PGP (19) and Ac-PGP (20), respectively. Recently, our group has characterized the presence of PGP peptides in patients with ALI, both in the systemic circulation (7) and in the lung (21). However, it remains unknown how Ac-PGP peptides are regulated in acute inflammation. Here, we present a potentially new mechanism of active transport of Ac-PGP tripeptide matrikines via the oligopeptide transporter PEPT2 across type II alveolar epithelial cells and identify this transporter as a modulator of Ac-PGP–dependent inflammation in ALI.

## Results

### Concentration of labeled Ac-PGP over time and neutrophilic inflammatory response in the lung.

**Figure 1:**
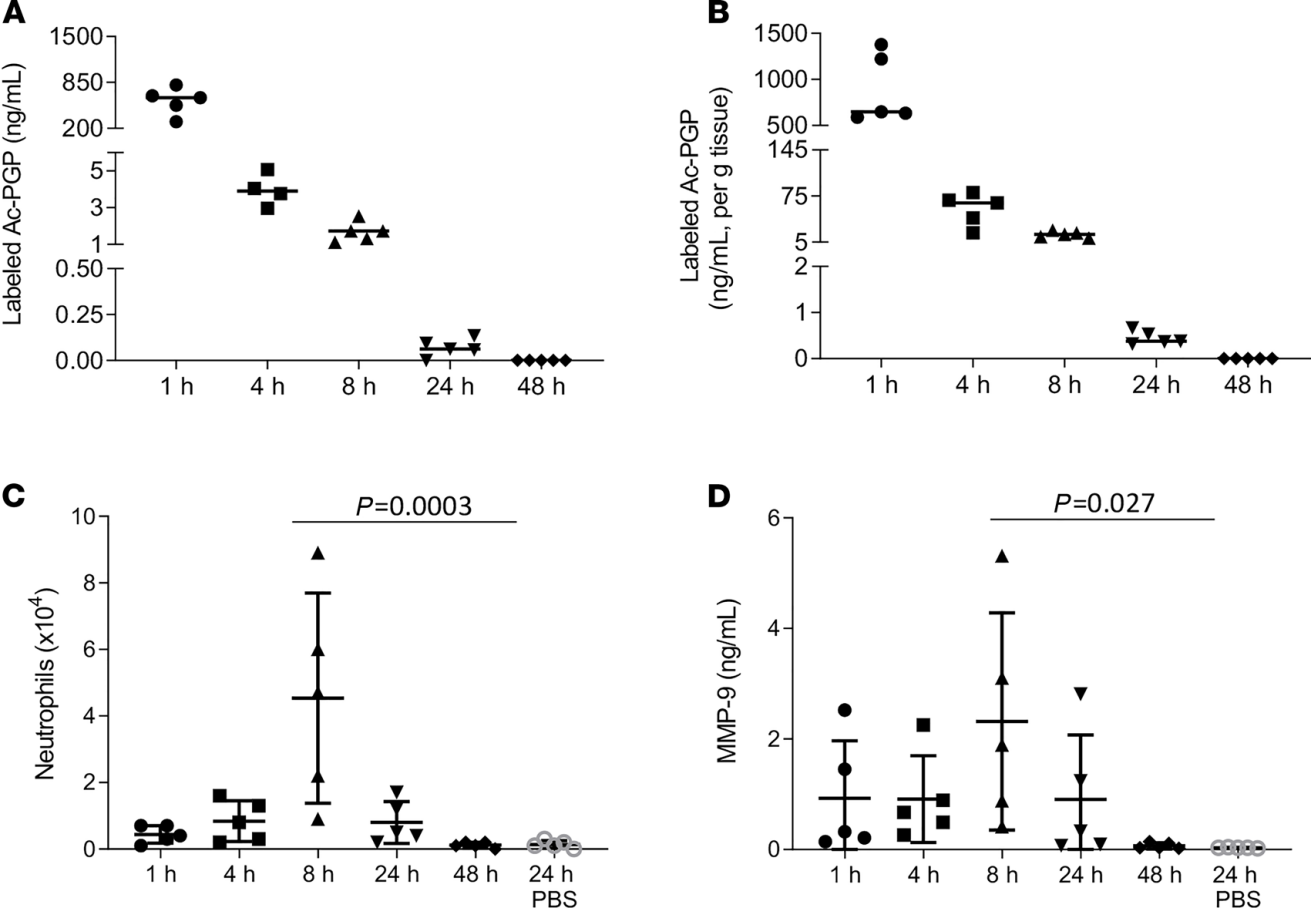
The measurement of labeled Ac-PGP distribution and labeled Ac-PGP–induced airway neutrophilic inflammatory response. Labeled Ac-PGP peptide (250 μg in 50 μl) was intratracheally administered to 6- to 8-week-old C57BL/6J mice (*n* = 5 animals per time point). The bronchoalveolar lavage (BAL) (**A**) and lung tissue (**B**) was collected at multiple time points after treatment for the measurement of labeled Ac-PGP by electrospray ionization–liquid chromatography–tandem mass spectrometry (ESI-LC-MS/MS). The neutrophil cell counts (**C**) and MMP-9 level (**D**) were determined in BAL samples. Data are presented as the mean ± SD and were analyzed by 1-way ANOVA and Tukey’s multiple comparisons post test.

We utilized a C^13^N^15^-labeled acetylated PGP (labeled Ac-PGP) peptide to distinguish delivered versus endogenously generated Ac-PGP (22). Labeled Ac-PGP peptide was intratracheally administered to mice, and bronchoalveolar lavage (BAL) was collected at various time points after treatment. The concentration of labeled Ac-PGP was high in the BAL at 1 hour, with a rapid decline in concentration by 4 hours (Figure 1A). In addition to BAL, lung and peripheral tissues were collected at sequential time points after intratracheal administration of labeled Ac-PGP (Supplemental Table 1; supplemental material available online with this article; https://doi.org/10.1172/jci.insight.140750DS1). One hour after treatment, labeled Ac-PGP was detected in substantial quantities in the lung (Figure 1B) and in the distal organs (Supplemental Table 1). The BAL fluid was further analyzed for evidence of neutrophilic inflammation. As expected, the animals that received labeled Ac-PGP had increased neutrophil recruitment into the lung (Figure 1C). MMP-9, a critical neutrophil-derived proteolytic enzyme that catalyzes intermediate steps of collagen breakdown to PGP peptides, was also increased in the BAL following labeled Ac-PGP administration (Figure 1D). These results show that after intratracheal administration, Ac-PGP peptides redistribute systemically, providing critical insight to normal matrikine distribution in vivo. Further, the translocation of Ac-PGP from the airway of these animals suggests active transport of PGP peptides into the systemic compartment.

### PEPT2 expression and in vitro transport of Ac-PGP in human distal epithelial cells.

**Figure 2:**
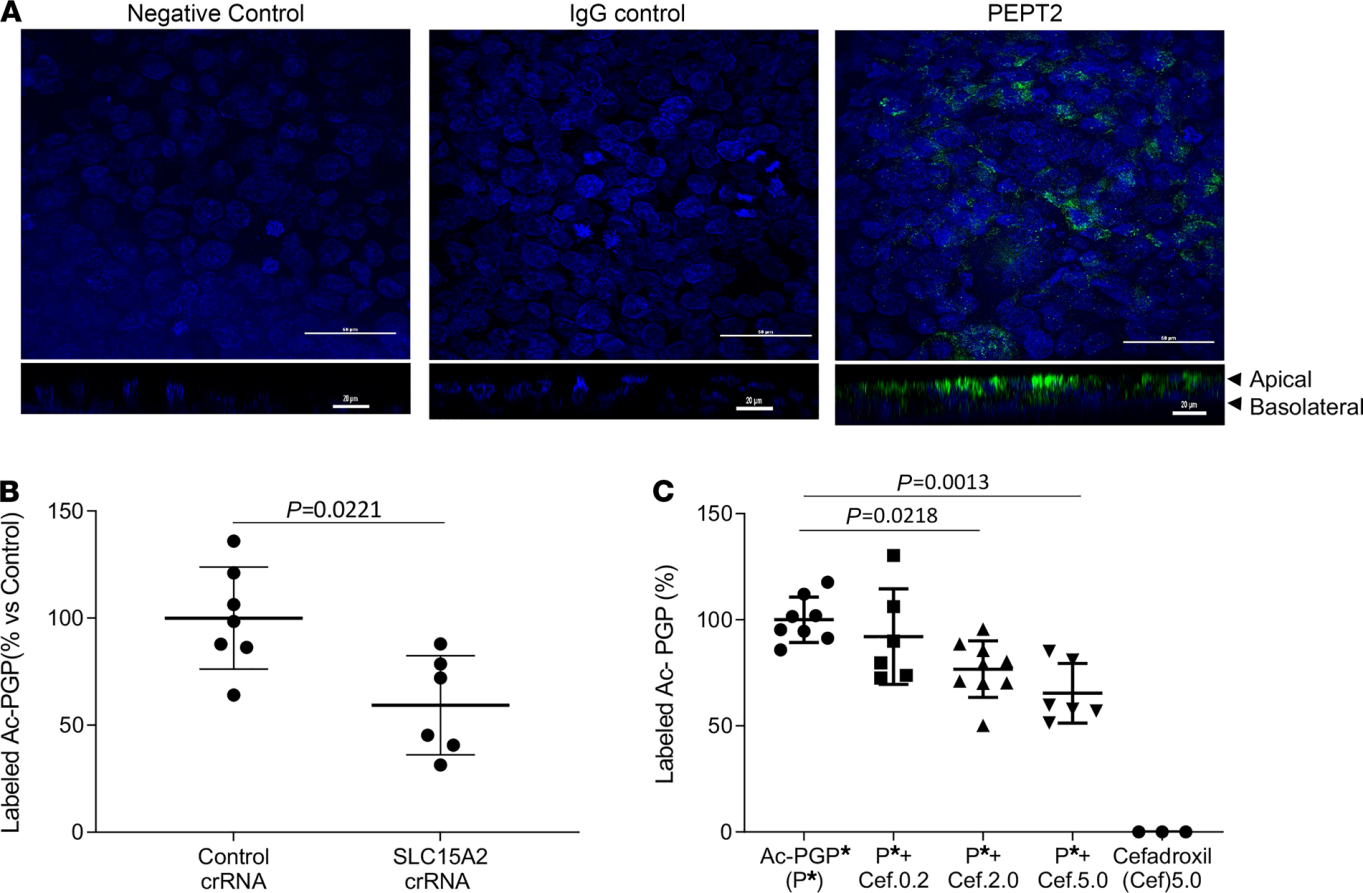
The transport of Ac-PGP in human distal lung epithelial cell line NCl-H441 was inhibited by blocking PEPT2. The expression of PEPT2 protein (green fluorescence) in polarized NCl-H441 (H441) cells (nuclei, blue fluorescence) was examined by immunohistological staining. Scale bar: 50 μm. *Z*-stacking images are shown below. Scale bar: 20 μm (**A**). The transepithelial transport of labeled Ac-PGP (50 ng/ml) in the apical-to-basal directions across PEPT2-knockdown H441 cell monolayers was measured by ESI-LC-MS/MS (**B**). The transepithelial transport of labeled Ac-PGP (Ac-PGP*, 50 ng/ml) in the apical-to-basal directions across H441 cell monolayers was measured in the presence of PEPT2 inhibitor cefadroxil (0.2 mM, 2.0 mM and 5.0 mM; Cef.0.2, Cef.2.0, and Cef.5.0, respectively) by ESI-LC-MS/MS (**C**). Data (mean ± SD) are expressed with 3–9 wells per group and were analyzed by Mann-Whitney test or 1-way ANOVA with Tukey’s multiple comparisons post test. crRNA, CRISPR RNA.

We next investigated the mechanism by which this matrikine was actively transported from the lung. We focused on the solute carrier 15 (SLC15) family of the proton oligopeptide transporters, effective transporters of di- and tripeptides (23, 24). Specifically, the PEPT2 transporter has previously been shown to be expressed on the apical surface of type II alveolar cells (25–27), although its role in the lung has remained poorly understood. We cultured and polarized the human distal lung epithelial cell line NCI-H441 (H441) and validated apical PEPT2 expression via immunofluorescence (Figure 2A). We performed in vitro SLC15A2 gene silencing in H441 cells to examine whether knockdown of SLC15A2 would prevent PEPT2-mediated Ac-PGP peptide transport. When SLC15A2 was silenced in H441 polarized cells by CRISPR/Cas9 SLC15 A2 CRISPR RNA (Supplemental Figure 1, A and B), labeled Ac-PGP transport across the H441 monolayer from apical to basolateral was significantly decreased, suggesting a unique mechanism involving SLC15A2 transport of Ac-PGP peptides (Figure 2B). Similar results were obtained using the potent PEPT2 competitive inhibitor cefadroxil (27). Pretreatment with cefadroxil significantly reduced, in a dose-dependent manner, the amount of labeled Ac-PGP transported across the epithelial cell monolayer (Figure 2C). Interestingly, cefadroxil did not alter the activity of the enzymes that generate Ac-PGP (Supplemental Figure 2, A and B), suggesting that this effect is related to peptide redistribution. These results provide clear evidence of the capability of PEPT2 to serve as an active transporter of Ac-PGP in the distal airway epithelia.

### In vivo transport of Ac-PGP with PEPT2 inhibitor augments lung matrikine accumulation.

**Figure 3:**
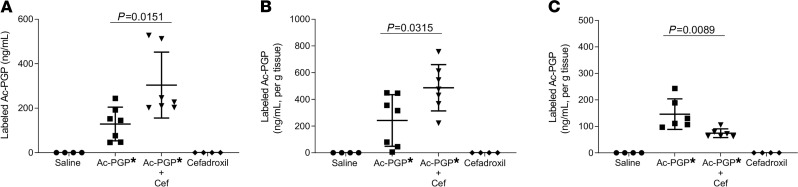
Ac-PGP uptake in the lung was inhibited by blocking PEPT2. Labeled Ac-PGP peptide (Ac-PGP*, 250 μg in 50 μl saline) was intratracheally administered to C57BL/6J female mice 30 minutes after intratracheal pretreatment with PEPT2 inhibitor cefadroxil (Cef, 250 μg/mouse). The concentration of labeled Ac-PGP in BAL (**A**), lung tissue (**B**), and pulmonary window tissue (**C**) was measured by ESI-LC/MS/MS. Data (mean ± SD) are expressed with 4–7 mice per group and were analyzed by 1-way ANOVA and Tukey’s multiple comparisons post test.

Our distribution experiments show that following intratracheal instillation of labeled Ac-PGP, the labeled tripeptide was detected in peripheral tissues. In vitro studies suggested the central role of PEPT2-based transport as critical to this effect. To investigate the in vivo transport of Ac-PGP from the lung into systemic circulation via PEPT2, mice were pretreated with PEPT2 inhibitor cefadroxil before intratracheal administration of labeled Ac-PGP. BAL, lung, and peripheral tissues were collected. The animals that received labeled Ac-PGP plus cefadroxil had higher levels of labeled Ac-PGP in BAL and lung tissue (Figure 3, A and B, respectively) and lower levels in the pulmonary window (Figure 3C), the most proximal extrapulmonary site, compared with the animals that received labeled Ac-PGP only. We also observed that the animals treated with labeled Ac-PGP plus cefadroxil had increased levels (78.41 ± 41.30 ng/mL) of activity of the PMN surrogate myeloperoxidase (MPO) in the lung tissue compared with animals that received labeled Ac-PGP alone (41.17 ± 12.99 ng/mL, *P* = 0.035 via Mann-Whitney test). This coincides with having higher levels of labeled Ac-PGP in the lung tissue. Therefore, inhibition of the PEPT2 transporter led to accumulation of Ac-PGP in the lung and, subsequently, an increase in neutrophilic inflammation in vivo. These results further demonstrate that Ac-PGP peptides are a substrate of PEPT2 and inhibition of that transporter caused pulmonary accumulation and reduced systemic distribution of this matrikine.

### PEPT2 inhibition increased LPS-induced acute lung inflammation and matrikine accumulation.

**Figure 4:**
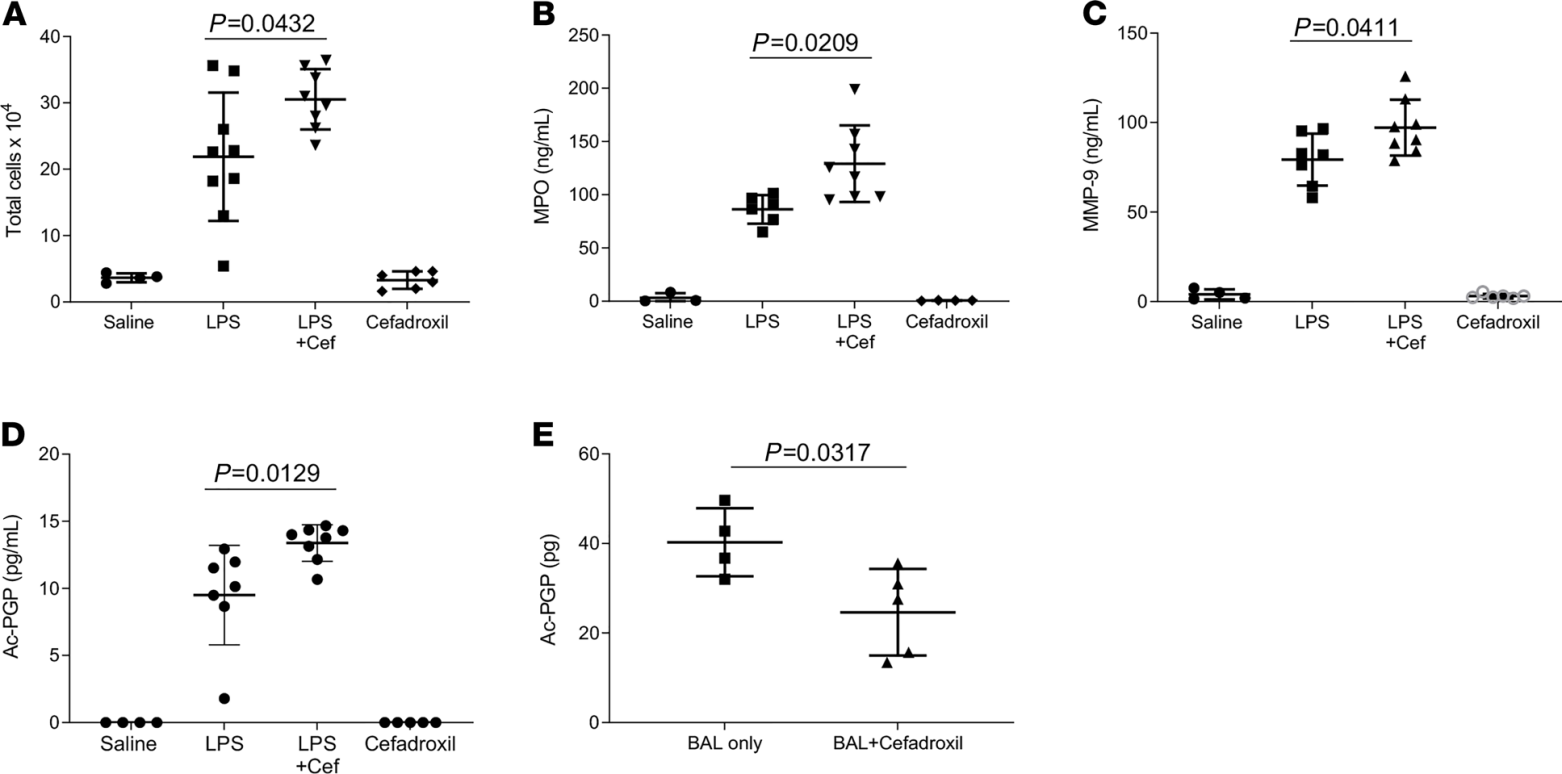
The application of PEPT2 inhibition increased LPS-induced acute lung inflammation and blocked Ac-PGP transport in patients with ARDS. C56BL/6J mice were intratracheally (i.t.) treated with cefadroxil (Cef) and 100 μg LPS (*P*. *aeruginosa*). BAL was collected after treatment for total cell counts (**A**), MPO (**B**), and MMP-9 (**C**) levels. The level of Ac-PGP peptides in the BAL was analyzed by ESI-LC-MS/MS (**D**). Statistical analysis was performed using 1-way ANOVA and Tukey’s multiple comparison post test, *n* = 3–9 mice/group. All values represent mean ± SD. The transepithelial transport of Ac-PGP peptides in BAL of patients with ARDS in the apical-to-basal directions across polarized H441 cell monolayers was measured with or without cefadroxil by ESI-LC-MS/MS (**E**). Data (mean ± SD) are expressed with 4–5 wells per group and were analyzed by Mann-Whitney test.

Previous studies from our lab showed that PGP peptides are involved in neutrophil recruitment in LPS-induced ALI (21). Ac-PGP transport from the lung was investigated in the setting of ALI using a LPS-induced ALI model. There was an increased total cell count and increased MPO and MMP-9 activity in the BAL of the LPS-challenged mice treated with cefadroxil, consistent with the expected neutrophilic inflammatory response to LPS-induced lung injury (Figure 4, A–C). BAL fluid analysis also showed increased Ac-PGP levels in LPS-treated mice that also received cefadroxil (Figure 4D). We show that, utilizing a model of ALI, inhibition of the PEPT2 transporter increases matrikine levels in the lung and subsequently increases inflammatory response.

### Transport of endogenous Ac-PGP in BAL of patients with ARDS across distal lung epithelial cells is attenuated by PEPT2 inhibitor.

To evaluate the transepithelial transport of endogenous Ac-PGP generated in patients with ALI, a pooled BAL sample from patients diagnosed with ARDS (21) was applied to a cultured monolayer of distal human H441 epithelial cells. Cefadroxil was added to the apical surface of the H441 cell monolayer before the ARDS BAL fluid and then media from the basolateral compartment was collected for analysis. There were significantly lower levels of Ac-PGP in the basolateral fluid of cells pretreated with cefadroxil compared with saline controls (Figure 4E). These results demonstrate that Ac-PGP in biological specimens is capable of being actively transported from the apical to basolateral surface via PEPT2.

## Discussion

To our knowledge, this is the first study to determine the in vitro and in vivo significance of PEPT2 on the distribution of critical lung matrikines, Ac-PGP peptides. Following intratracheal administration of labeled Ac-PGP in a murine model, there was a rapid decline in concentration of the labeled peptide in the BAL over time. Simultaneously, significant levels of labeled Ac-PGP were detected in multiple organs, suggesting active transport from lung epithelium to the systemic compartment of these animals. We focused our investigation on the SLC15 family of proton oligopeptide transporters. These transporters mediate the uptake of di- and tripeptides by coupling substrate translocation to a proton gradient (23, 24). PEPT1 (also known as SLC15A1) is a transporter in this group that is primarily expressed in the intestine. Under normal physiologic conditions it transports di/tripeptides for metabolic purposes. However, in a chronic inflammatory disease state, such as inflammatory bowel disease, the PEPT1 transporter is upregulated and small bacterial peptides are transported into enterocytes where they interact with innate immune receptors and activate inflammatory pathways (28). PEPT2 is expressed in the mammary gland epithelium, the choroid plexus, renal tubular cells, and the lung (25, 29–31). PEPT2 has been shown to play a role in neuropeptide homeostasis in the CSF (29). In the lung, PEPT2 is expressed in the alveolar II pneumocytes, bronchial epithelium, and small vessel endothelium (25). Based on the physiological function of this family of transporters to maintain homeostasis and also propagate inflammation in a disease state as well as the tissue expression of this particular transporter, we focused on PEPT2 as a potential target for Ac-PGP.

A unique feature of this group of transporters is the diversity of substrates because substrate affinity is unaffected by peptide charge (31, 32). To evaluate peptide transport in our experiments, we used the first-generation cephalosporin antibiotic cefadroxil as a competitive inhibitor of PEPT2 transporter. Cefadroxil has been studied as a target substrate for the PEPT2 transporter in studies evaluating renal transport and drug reabsorption (33). PEPT2 has a much higher affinity for most substrates, especially cefadroxil, compared with other proton oligopeptide transporters. One group described the inhibitor constant of PEPT2 for cefadroxil as 3 μM compared with 2170 μM for PEPT1 (34). Considering the high substrate affinity of PEPT2 for cefadroxil and the absence of PEPT1 expression in our target tissue population (35), cefadroxil is a reasonably specific and effective transporter inhibitor. The animals that received labeled Ac-PGP plus a PEPT2 inhibitor, cefadroxil, had higher levels of Ac-PGP in BAL and lung tissue and lower levels in the peripheral tissues compared with the animals that received labeled Ac-PGP only. This suggests that Ac-PGP peptides may be a substrate of PEPT2 and inhibition of that transporter caused pulmonary accumulation of this matrikine. When mice were treated with cefadroxil, the transport of exogenous Ac-PGP was blocked and Ac-PGP accumulated in the lung, which augmented the inflammatory response. This phenomena was confirmed in the disease model of ARDS, with enhanced inflammation due to PEPT2 inhibition.

We further investigated PEPT2/SLC15A2 as a transporter for Ac-PGP through in vitro studies using human distal lung epithelial cells (H441 cells). These cells have an alveolar type II phenotype, and they can form epithelial-like monolayers (27, 36). By applying cefadroxil to the airway epithelial cells, the transport of Ac-PGP across cell layers was suppressed from the apical to the basolateral side. Furthermore, blocking the oligopeptide transporter, PEPT2, when ARDS BAL fluid, containing endogenous Ac-PGP peptides, was applied to the apical surface led to lower levels of Ac-PGP in the basolateral compartment compared with samples with no inhibitor. These results demonstrate that active transport via an oligopeptide transporter may be a mechanism for matrikine regulation in the lung.

This work builds on prior literature demonstrating the importance of peptide transporters in modulating human disease. Specifically, members of the SLC15 family, which are present on the mucosal surfaces of the GI tract and have been associated with inflammatory bowel disease (24). Importantly, the expression of these transporters in the kidney has a tremendous effect on antibiotic concentrations and associated toxicities in vivo (37). Our work provides key data about the importance of these transporters in the lung but now provides a critical role for the regulation of endogenous bioactive peptides regulating neutrophilic lung disease. Our data establish a critical role for PEPT2 in the regulation of Ac-PGP peptides in the inflamed lung. The active transport of these matrikines from lung to systemic circulation suggests that PEPT2 plays an important role in modulating the physiological, pharmacological, and pathological activities of endogenous matrikines in ARDS.

The purpose of this transporter in regulating Ac-PGP in acute lung inflammation is not clearly known. One possibility is that when the burden of Ac-PGP overwhelms ACE degradation abilities (20) and passive diffusion, the transporter can redistribute the peptide to attenuate inflammatory response. This may be supported by the fact that PEPT2 requires a proton gradient for peptide transport (38). Another possibility is that the redistribution of Ac-PGP to the basolateral surface during acute inflammation may lead to compartment-specific signaling critical for modulating host response.

ARDS is traditionally associated in the medical ICU with sepsis and bacterial or viral pneumonia (9). The salient nature of this work has become more evident as SARS-CoV-2 has emerged as a pathogen leading to progressive respiratory failure and ARDS, a frequent clinical manifestation with increased disease morbidity and mortality (39–42). The findings in this study may have implications in understanding the pathophysiology and developing therapeutic targets, as neutrophilia is a key feature in patients with COVID-19 (43). Therefore, the identification of this regulatory pathway of neutrophilic inflammation may provide new therapeutic targets to modulate lung inflammation and damage in devastating disorders such as COVID-19–related ARDS.

## Methods

### Materials.

A C^13^N^15^-labeled Ac-PGP (heavy label on both prolines, labeled Ac-PGP) was used to distinguish C^13^N^15^-labeled Ac-PGP from Ac-PGP generated endogenously from collagen. Labeled Ac-PGP was synthesized by Bachem Americas Inc. *Pseudomonas aeruginosa–*derived LPS was purchased from MilliporeSigma. MMP-9 ELISA kits were purchased from R&D Systems Inc. MPO ELISA kits were purchased from Calbiochem. IgM ELISA kits were purchased from the Immunology Consultants Laboratory. The H441 cell line (HTB-174) and RPMI medium were purchased from ATCC. Dexamethasone and insulin-transferrin-sodium selenite were purchased from MilliporeSigma and Roche, respectively. Cefadroxil was purchased from MilliporeSigma. Actin antibody was purchased from MilliporeSigma, and PEPT2 antibody was purchased from Abcam (ab83771).

### Mass spectrometry.

Ac-PGP and C^13^N^15^-labeled Ac-PGP peptides from the in vivo and in vitro experiments were quantified using electrospray ionization–liquid chromatography–tandem mass spectrometry (ESI-LC-MS/MS) as we have previously published (13, 22). Spectra from Ac-PGP show a total peptide mass-to-charge ratio (*m/z*) of 312, with daughter ions of 112 and 140 m/z. This is compared to spectra from C13N15 Ac-PGP, which show a total peptide *m/z* of 324, with daughter ions at 117 and 146 *m/z*. Peak area was measured and labeled Ac-PGP peptide concentrations were calculated using a relative standard curve method as previously described (22).

### Murine model.

C56BL/6J wild-type female mice were used for all animal experiments. All animals were cared for according to the University of Alabama at Birmingham (UAB) Institutional Animal Care and Use Committee. To evaluate PGP-induced local inflammation and systemic distribution, mice were anesthetized with inhaled 5% isofluorane followed by intratracheal instillation of labeled Ac-PGP (250 μg in 50 μL). Control mice were treated with intratracheal instillation of an equal volume of PBS. The mice were euthanized at various time points following treatment. BAL serum and tissue (pulmonary window, lung, heart, liver, spleen, brain, and kidney) were collected for measurement of labeled Ac-PGP by ESI-LC-MS/MS. Ac-PGP concentration in tissue is expressed as ng/mL per gram of tissue to account for the size of organs/tissue in individual mice. BAL was also analyzed for neutrophil count and by substrate-specific ELISA for MMP-9. For peptide transporter 2 (PEPT2) inhibitor studies, mice were pretreated with intratracheal instillation of cefadroxil (250 μg) 30 minutes before administration of labeled Ac-PGP. The animals were then euthanized at 1 hour following treatment for collection of BAL and tissue samples. The concentration of labeled Ac-PGP in BAL and tissue was measure with mass spectrometry. MPO level in the lung tissue was measured by ELISA assay.

To create an LPS-induced ALI model, we used a well-established protocol used by our lab in previous studies (21). Mice were anesthetized with 5% isofluorane followed by intratracheal instillation of LPS (100 μg in 50 μL). Control mice were treated with intratracheal instillation of an equal volume of saline. For PEPT2 inhibitor studies, mice were pretreated with intratracheal administration of cefadroxil (500 g) 30 minutes before LPS. These mice also received an additional 4 doses of intratracheal cefadroxil (500 g). Forty-eight hours after LPS exposure, the mice were euthanized for collection of BAL and tissue. BAL was collected from euthanized mice by instillation of 1 mL of PBS through a tracheal cannula. The returned fluid was centrifuged at 400*g* for 10 minutes at 4°C. The supernatant was saved for ELISA studies. The cell pellet was used for cell count via hemocytometer. Animal tissue was collected from euthanized mice and immediately frozen in liquid nitrogen. The tissue was prepared for mass spectrometry and ELISA analysis by homogenization of the tissue in 500 μL sterile PBS using Qiagen TissueLyser II and 5 mm stainless steel beads. Tissue homogenate was centrifuged at 13,000*g* for 15 minutes at 4°C, and supernatant was filtered by 10,000 MWCU with Amicon Ultra-0.5 centrifugal filter unit (MilliporeSigma).

### Cell culture.

H441 cells are human epithelial cells originally isolated from a patient with papillary adenocarcinoma of the lung, and they are an ideal cell model to study epithelial transport because they can form epithelial-like monolayers (36). H441 cells were cultured in RPMI-1640 medium with 10% FBS and penicillin/streptomycin (100 U/mL each) at 37°C in a 5% CO_2_ incubator. Twenty-four hours after seeding, the medium was replaced with RPMI-1640 medium with additional supplements. In addition to the FBS and penicillin/streptomycin, 200 nM dexamethasone and insulin-transferrin-sodium were added. This complete medium was used to maintain cell cultures for a total of 13 days after seeding, changing medium every 48 hours.

### Transepithelial transport studies.

For transepithelial transport studies, H441 cells were seeded at a density of 2.23 × 10^5^ cells/cm^2^ on Costar Transwell Permeable Supports (6.5 mm diameter, pore size 04 μm). Apical and basolateral fluid volumes were 0.2 mL and 0.6 mL, respectively. Twenty-four hours after seeding, the apical medium was removed and the medium in the basolateral chamber was replaced with complete medium. PEPT2 was knocked out of H441cells by the CRISPR/Cas9 system. Transport studies with cell monolayers were conducted on day 13. MES buffer (pH6.0) containing D-glucose (5 mM) and labeled Ac-PGP (50 ng) was added to apical compartment, and the cells were incubated at 37°C. Samples were collected from the basolateral compartment to measure labeled Ac-PGP concentration by mass spectrometry. To examine the effect of PEPT2 inhibition, cell monolayers were pretreated with cefadroxil, a potent inhibitor of PEPT2, at different concentrations (0.2 mM, 2.0 mM, and 5.0 mM).

### Human subjects.

A previous study from our laboratory evaluated Ac-PGP levels in BAL from patients admitted to the ICU with Gram-negative sepsis ARDS. PGP levels were measured and found to be higher in patients with ARDS compared with mechanically ventilated patients without ARDS (21). Samples of ARDS BAL from this study, with previously determined Ac-PGP levels, were used in the transepithelial transport studies.

### Detection of PEPT2 protein by immunofluorescence.

H441 cells were cultured on Transwell membrane inserts for 13 days and then fixed on the membrane with –20°C 100% methanol. After blocking, the cells were incubated with 10 μg/mL anti-SLC15A2 antibody in 1% BSA in PBST (Abcam, ab83771) and subsequently incubated with secondary antibody DyLight 488 goat anti-rabbit IgG (1:250 dilution, Abcam, ab96899). A scalpel was used to release the membrane from the apparatus. The membrane was mounted on a glass slide using fluoroshield mounting medium with DAPI (Abcam, ab104139). Cells were visualized with a confocal microscope (Nikon C2).

### Statistics.

Data were analyzed and plotted using Prism 8 (GraphPad Software). Data are expressed as mean ± SD. Significant differences between control and treatment conditions in each experiment were analyzed by using unpaired, 2-tailed *t* tests or by an ANOVA followed by Tukey’s test for multiple comparisons. *P* < 0.05 was considered significant.

### Study approval.

C56BL/6J wild-type female mice were used for all animal experiments. All animals were cared for according to and with the approval of the UAB Institutional Animal Care and Use Committee (IACUC 120709133). Patients with ALI, secondary to Gram-negative sepsis, who were intubated and mechanically ventilated, were recruited from the UAB hospital (*n* = 4). All subjects carried the diagnosis of ARDS based on accepted diagnostic criteria (1). All human studies were approved by the UAB Institutional Review Board (protocol F081016007).

## Author contributions

SWR, RPP, AG, and XX provided conception and design. SWR, JL, LV, JPB, and XX conducted experiments. SWR, JL, LV, JPB, JEB, AG, and XX provided analysis and interpretation. SWR, AG, XX, and RPP drafted the manuscript for important intellectual content.

## Supplementary Material

Supplemental data

## Version 1 04/08/2021

Electronic publication
