# Supplementary material for: A mechanism for matrikine regulation in acute inflammatory lung injury
Source: JCI Insight. 2021 Apr 8;6(7):e140750. doi: 10.1172/jci.insight.140750 (PMC8119180; doi:10.1172/jci.insight.140750)
Supplement: Supplemental data [file jciinsight-6-140750-s184.pdf]

A Mechanism for Matrikine Regulation in Acute Inflammatory Lung Injury

Sarah W Robison, JinDong Li, Liliana Viera, Jonathan P Blackburn, Rakesh P Patel, J Edwin Blalock, Amit Gaggar, Xin Xu

SUPPLEMENT MATERIAL

FIGURES

Supplemental Table S1. The labeled Ac-PGP was distributed in the blood and different organs after intratracheal instillation

Time	Serum labeled Ac-PGP (ng/ml) (SD)	Labeled Ac-PGP (ng/ml, per g tissue)(SD)					
		PW	Heart	Kidney	Spleen	Liver	Brain
**1 h**	48.29 (15.68)	1583.42 (777.10)	41.78 (28.01)	343.61 (111.03)	14.23 (8.81)	11.34 (7.37)	4.1 (3.6)
**4 h**	0.62 (0.14)	87.41 (41.93)	2.00(0.06)	5.68 (2.49)	1.58 (0.72)	0.45 (0.39)	0.14 (0.12)
**8 h**	0.36 (0.13)	28.47 (12.55)	0.44 (0.01)	4.37 (1.95)	0.98 (0.67)	0.47 (0.81)	0.13 (0.24)
**24 h**	0	0.69 (0.03)	0	0.85 (1.53)	0	0	0
**48 h**	0	0.00	0	0	0	0	0

Note: Different organs were collected at multiple time points after treatment for the measurement of labeled Ac-PGP levels by ESI-LC-MS/MS. PW=pulmonary artery window, collection of hilar tissue including aortic arch, pulmonary artery, lymph nodes and fatty tissue.

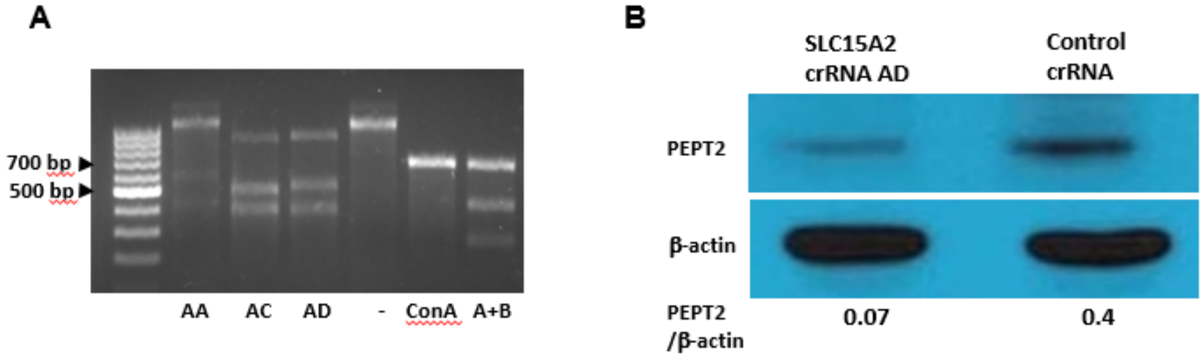

Supplemental Figure S1. The knockdown of PEPT2 in H441 cells. Polarized H441 cells were transfected with SLC15A2 crRNA using CRISPR-Cas9 system. Genomic DNA from CRISPR-Cas9–edited SLC15A2 (AA, AC and AD) in H441 cells were PCR amplified, digested using T7 endonuclease I, and run on the gel **(A)**. Reference standard bands at 1000 bp (upper marker) and 100 bp (lower marker) are used to align the gel and analyze the results. Estimated band sizes for the cut and uncut positive control amplicons are shown in the gel image. ConA contains homoduplexes of Control A PCR products, while A+B contains homoduplexes and heteroduplexes of Control A and B PCR products. The levels of PEPT2 expression was determined by western blot analysis of lysates from transfected H441 cells with actin controls **(B)**. Densitometric analysis of PEPT2 immunoblotting data normalized against actin with the control was shown beneath the image

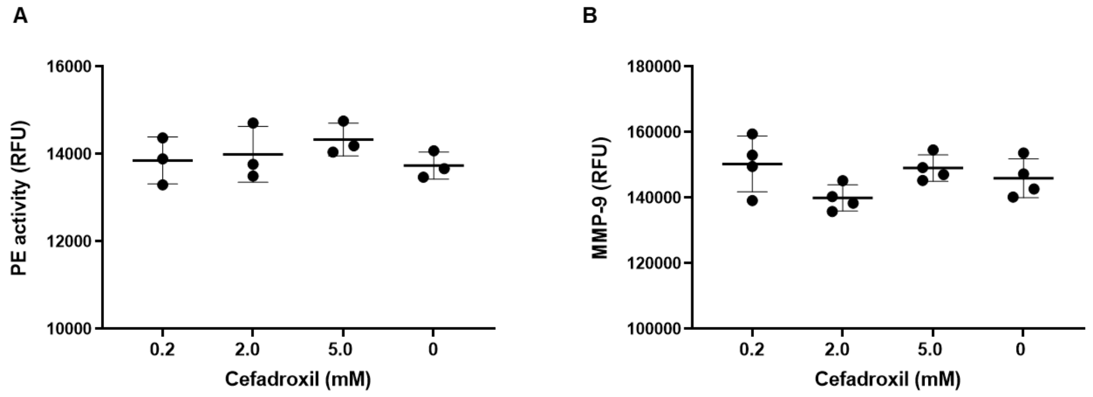

Supplement Figure S2. Cefadroxil dose not affects enzyme activity. Cefadroxil was preincubated with rPE (12.5 nM) for 60 minutes at varying concentrations before the addition of a specific flurogenic substrate Suc-Gly-Pro-7-amido-4-methylcoumarin (Suc-Gly-Pro-AMC) (Bachem, Bubendorf, Switzerland). The cleavage of AMC from substrate by rPE was detected using a Fluometer Reader at an excitation wavelength of 360 nm and an emission wavelength of 460 nm **(A)**. Cefadroxil was preincubated with rMMP-9 (16 ng/ml) for 60 minutes at varying concentrations before the addition of a fluorogenic substrate (Fluor-Pro-Leu-Gly-Leu-Ala-Arg-NH2). The quantification of MMP-9 activity by ELISA-based assay (Cat No F9M00, R&D Systems, Minneapolis, MD) using a Fluometer Reader at an excitation wavelength of 320 nm and an emission wavelength of 405 nm **(B)**. Data are presented as the mean ± SD and analyzed by Tukey’s multiple comparisons post-test.
